# Syringin inhibits endogenous volume regulated anion channel currents of HEK293 cells in hypotonic circumstances

**DOI:** 10.1002/prp2.1105

**Published:** 2023-06-06

**Authors:** Ziwei Xu, Wei Yao, Minyan Liang, Song Wang, Longhui Lu, Jingjing Wang, Na Zhu, Liping Huang

**Affiliations:** ^1^ School of Pharmacy Jiangxi University of Chinese Medicine Nanchang P. R. China

**Keywords:** inhibitor, molecular docking, syringin, VRAC channels

## Abstract

Syringin is a natural chemical compound first isolated from the bark of lilac and is known to have neuroprotective effects in middle cerebral artery occlusion (MCAO). Volume regulated anion channel (VRAC) is a cell swelling‐activated anion channel, which is implicated in brain ischemia. However, the mechanism underlying the syringin protecting the neuron from damage in MCAO is still unclear. We hypothesized that syringin has an inhibitory effect on the opening of VRAC channels. To access the effect of syringin on VRAC currents and predict how syringin interacts with VRAC proteins, we performed whole‐cell patch‐clamp experiments using HEK293 cells. Initially, HEK293 cells were perfused with isotonic extracellular solution, followed by hypotonic extracellular solution to stimulate endogenous VRAC currents. Once the VRAC currents reached a steady state, the hypotonic solution containing syringin was perfused to study the effect of syringin on VRAC currents. The potential interaction between syringin and the VRAC protein was investigated using molecular docking as a predictive model. In this study, we found that syringin moderately inhibited VRAC currents in a dose‐dependent manner. The potential binding of syringin to LRRC8 protein was predicted through in silico molecular docking, which suggests an affinity of −6.6 kcal/mol and potential binding sites of arginine 103 and leucine 101. Our results herein characterize syringin as an inhibitor of the VRAC channels, which provides valuable insights for the future development of VRAC channel inhibitors.

## Graphical abstract

To record wild‐type volume regulated anion channel (VRAC) currents, HEK293 cells were plated on glass coverslips and treated with a cellular hypotonic solution. The inhibitory effect of syringin on VRAC currents was tested using patch‐clamp techniques. Results showed that syringin inhibited VRAC channel activity.

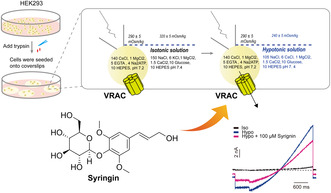

**Abbreviations**
EC: extracellular domain
IL: intracellular linker domain
LRR: leucine‐rich repeat domain
MCAO: middle cerebral artery occlusion
ROS: reactive oxygen species
RVD: regulatory volume decrease
VRAC: volume regulated anion channel

## 1 INTRODUCTION

Volume regulated anion channel (VRAC) is an anion channel ubiquitously expressed in the membranes of vertebrate cells. Changes in osmotic pressure can cause cells to swell, and the process of regulatory volume decrease (RVD) is responsible for restoring the abnormal cell volume. VRAC channels play a critical role in RVD by facilitating the regulated passive efflux of chloride and small organic compounds, thereby enabling water to exit the cytoplasm.^1^ In addition to transferring the inorganic ion and organic osmolytes, VRAC channel also mediates glutathione, cGAMP, and anti‐cancer drugs.^2^, ^3^, ^4^ VRAC channel has been identified as a heteromeric complex composed of LRRC8 protein family (LRRC8A, LRRC8B, LRRC8C, LRRC8D, and LRRC8E). Of these proteins, LRRC8A is the sole essential subunit required for functional VRAC channel formation, but it must also associate with at least one of the other LRRC8 proteins to form a functional channel.^5^, ^6^ The hexameric assembly of LRRC8 proteins results in a diverse range of VRAC complexes with different subunit compositions.^5^, ^7^, ^8^ High‐resolution cryo‐EM structures for homomeric LRRC8A were revealed in 2018 by three distinct research teams.^9^, ^10^, ^11^ The LRRC8 protein contains 4 transmembrane domains (TM1–TM4), extracellular domain (EC), intracellular linker domain (IL), leucine‐rich repeat domain (LRR), N‐terminus and C‐terminus located inside the cell. The VRAC currents display characteristic features, including moderate outward rectification and inactivation tendency under cytoplasmic positive potentials.^12^, ^13^, ^14^

Cell swelling is relevant to ischemia‐induced brain damage. Astrocytes are susceptible to swelling in the central nervous system injury, and this process is mediated by the opening of VRAC channels. The activation of VRAC channels facilitates excitatory glutamate efflux from astrocytes, resulting in neuronal damage. Studies have shown that defects in astrocytic VRAC channels attenuate glutamate‐dependent neuronal excitability and protect mice from brain damage.^15^, ^16^ In addition to their role in regulating cellular swelling, VRAC channels have also been implicated in cell apoptosis, insulin secretion, and antiviral immunity.^1^, ^3^, ^17^ DCPIB is a commonly used specific inhibitor of VRAC channels, with an IC_50_ of 4.1 μM in CPAE cells.^18^ It has been proved that DCPIB has beneficial effects on reducing infarct size in middle cerebral artery occlusion (MCAO),^19^ preventing swelling‐induced shortening of guinea‐pig atrial action potential duration,^18^ and attenuating inflammatory response and neuronal injury in microglia.^20^ Although other potent inhibitors of VRAC channels, such as pranlukast, zafirlukast, and 6u are available,^21^, ^22^ fewer inhibitors of VRAC channels are derived from natural product sources.

Syringin (4‐[(1E)‐3‐hydroxyprop‐1‐en‐1‐yl]‐2,6‐dimethoxyphenyl β‐D‐glucopyranoside), also known as eleutheroside B, is a monosaccharide derivative that is abundantly found in Tinospora sinensis (Lour.) Merr., Tinospora crispa, and Eleutherococcus senticosus. Syringin exhibits various pharmacological properties, including anti‐oxidation, anti‐inflammation, and immune inhibition.^23^, ^24^, ^25^ It has been shown to reduce infarct volume and water content in MCAO animal models.^26^ However, it is not clear whether syringin regulates VRAC channels.

In the present study, we recorded the endogenous VRAC currents induced by hypotonic extracellular solution in HEK293 cells. We measured the VRAC currents before and after adding syringin at a potential of −80 mV. Additionally, we utilized molecular docking to predict the potential binding sites of the syringin.

## 2 MATERIALS AND METHODS

### 2.1 Reagents

Syringin (CAS: 118‐34‐3, HPLC >98%) was purchased from Herbpurify, dissolved in DMSO to make a stock solution, and stored at −20°C. Solutions were freshly prepared from stock solutions before each experiment and protected from light. The final concentration of DMSO was less than 0.1%.

### 2.2 Cell culture

HEK293 cells were cultured in DMEM (Solarbio) supplemented with 10% FBS (Hyclone) in flask T25 and maintained at 37°C with 5% CO_2_. When cells were cultured to 70%–80% confluence, HEK293 cells were split by trypsin–EDTA (Gibco) and replated onto HD coverslips coated with 0.1 mg/mL poly‐L‐lysine (Sigma‐Aldrich) for electrophysiological recording.

### 2.3 Electrophysiological recording

Volume regulated anion channel currents were recorded in the standard whole‐cell configuration at 15–25°C using EPC 10 USB patch‐clamp amplifier and PATCHMASTER NEXT software (HEKA, GER). The sampling rate was 20 kHz and digitally filtered at 2 kHz. Series resistance compensation was set to 60%–80%. The electrodes were pulled by SUTTER P‐1000 puller, and the resistance was maintained at 3–5 MΩ. In the patch‐clamp experiments, bathing solution perfusion was delivered by eight‐channel perfusion valve control system (Warner Instruments).

For VRAC currents whole‐cell recording, the isotonic extracellular solution contained (in mM) 150 NaCl, 6 KCl, 1 MgCl_2_, 1.5 CaCl_2_, 10 glucose, and 10 HEPES (pH 7.4; 320 mOsm). To elicit VRAC currents, cells were exposed to a hypotonic solution containing (in mM) 105 NaCl, 6 CsCl, 1 MgCl_2_, 1.5 CaCl_2_, 10 glucose, 10 HEPES (pH 7.4; 240 mOsm). Capillary glass electrodes were filled with solution containing 140 mM CsCl, 1 mM MgCl_2_, 5 mM EGTA, 4 mM Na_2_ATP, and 10 mM HEPES (pH 7.2 with NaOH, 290 mOsm).

The standard test protocol consisted of a 600 ms step to −80 mV followed by a 2600 ms ramp from −100 to +100 mV from a holding potential of −30 mV, applied at 1500 ms intervals. The full voltage protocols consisted of a 2000 ms step protocol from −120 to +120 mV in 20 mV increment from a holding potential of −80 mV applied every 5000 ms.

### 2.4 Molecular docking

The 3D structure of the VRAC protein (PDB number: 6NZW) was obtained from the RCSB PDB Protein Data Bank (https://www.rcsb.org/). The solvent and organic were removed from VRAC protein by PyMOL software. The 2D structure of syringin (PubChem CID: 5316860) was acquired from PubChem (https://pubchem.ncbi.nlm.nih.gov/). The energy of syringin was reduced by the MM2 method in Chem 3D 19.0. The charge was added to syringin by Autodock Vina MGLTools 1.5.6. software.^27^ DCPIB is a VRAC channel specific inhibitor, the binding pocket of which was wrapped up by the grid box (center_x = 199, center_y = 199, center_z = 135, size_x = 10, size_y = 10, size_z = 18). The molecular docking work of syringin and VRAC protein depends on the grid box. The energy range was set as 1. The potential interacting amino acid residues were predicted by PyMOL software.

### 2.5 Statistical analysis

Images were integrated by Adobe Illustrator CS5 software. The original data analysis software is Graphpad prism 7.0. All experimental data are presented as mean ± standard error (mean ± SEM).

### 2.6 Nomenclature of target and ligand

Key protein target and ligand in this article are hyperlinked to corresponding entries in http://www.guidetopharmacology.org, the common portal for data from the IUPHAR/BPS Guide to PHARMACOLOGY,^28^ and are permanently archived in the Concise Guide to PHARMACOLOGY 2019/20.^29^

## 3 RESULTS

### 3.1 The inhibition of syringin on VRAC channels

**FIGURE 1:**
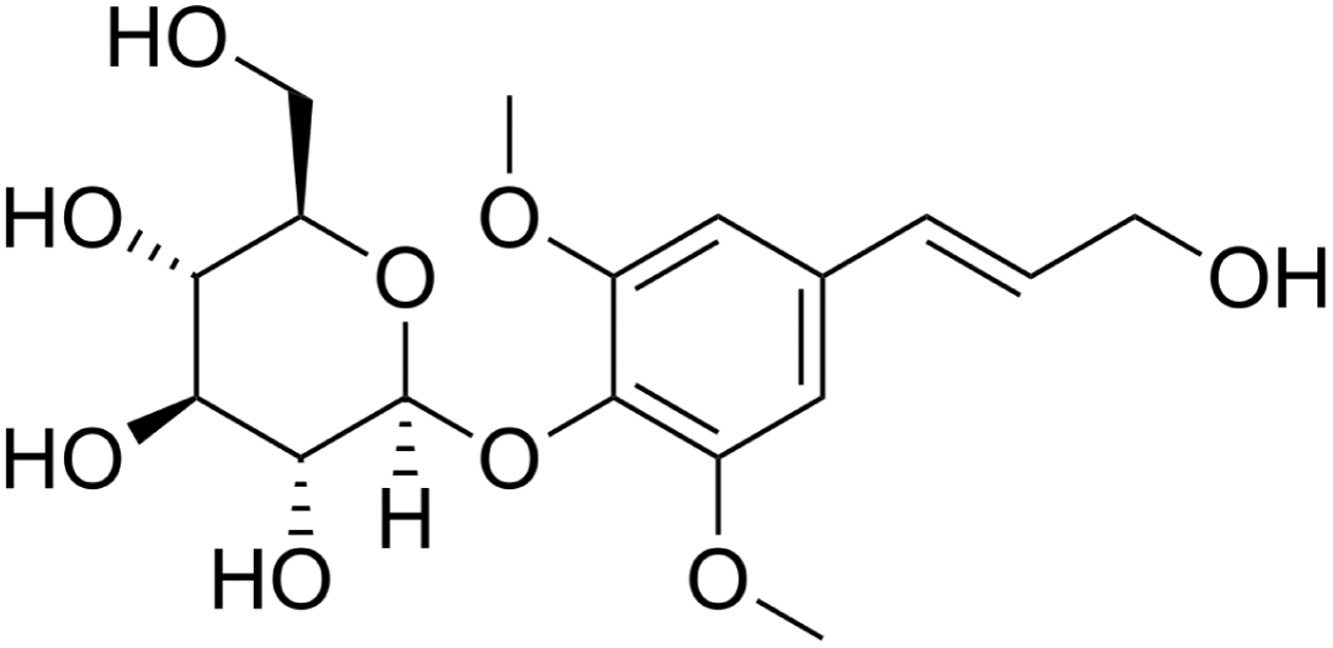
Chemical structure of syringin.

Syringin is a monosaccharide derivative, composed of trans‐sinapyl alcohol that is linked to a β‐D‐glucopyranosyl residue at position 1 through a glycosidic linkage (Figure 1). It has been found to have potential for rescuing MCAO animal models. However, the regulatory function of syringin on VRAC channels is still unknown.

**FIGURE 2:**
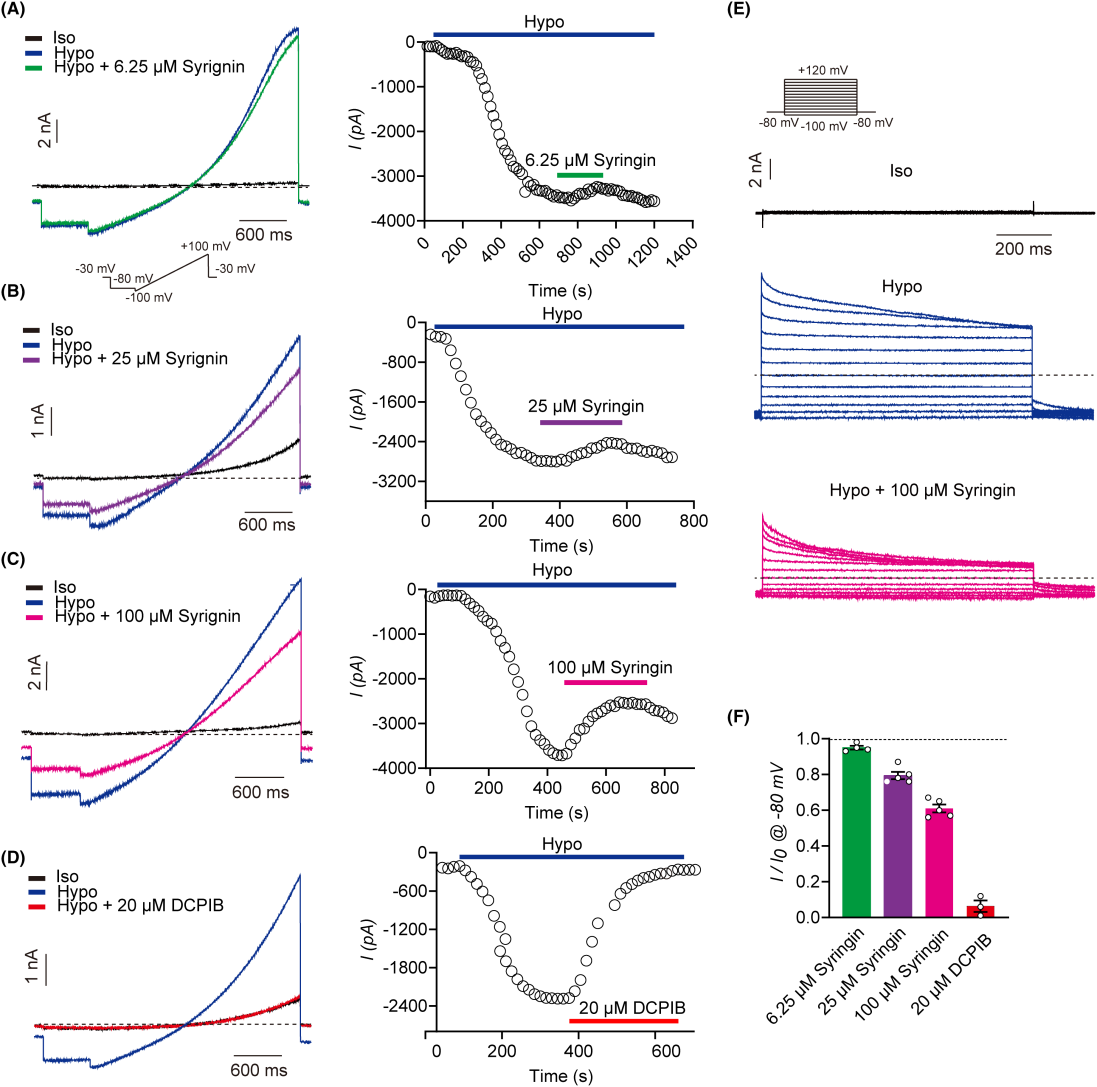
The inhibitory effect of syringin on volume regulated anion channel (VRAC) channels. (A–D) Typical current traces of VRAC in HEK293 (left). Currents were evoked by a 600 ms hyperpolarizing step of −80 mV from a holding potential of −30 mV, followed by a 2600 ms ramp protocol from −100 mV to +100 mV. And typical time courses of VRAC currents (right) were obtained at −80 mV with the application of syringin. (E) Representative traces of VRAC currents inactivation in response to voltage in isotonic solution (top), hypotonic solution (middle), and the application of 100 μM syringin (bottom), respectively. VRAC currents were obtained via a series of step protocols ranging from −100 mV to +120 mV in 20 mV increments from a holding potential of −80 mV and finally back to −80 mV. (F) Summary of the average inhibitory effect of syringin on VRAC channels at the 6.25 μM (*n* = 4), 25 μM (*n* = 5), and 100 μM (*n* = 5) tested at −80 mV. Each point represented an individual cell, results were mean ± SEM.

The patch‐clamp technique is considered the gold standard for assessing ion channel functionality. In order to investigate the effect of syringin on VRAC channels, we used the whole‐cell patch‐clamp method to measure changes in VRAC currents as a detection index. As VRAC channels are widely expressed in nearly every mammalian cell, we test the endogenous VRAC currents in the wild‐type HEK293 cell line. Initially, HEK293 cells were bathed in isotonic extracellular solution to maintain physiological osmosis pressure, in which no VRAC currents were observed. To elicit endogenous VRAC currents in HEK293 cells, we perfused them with hypotonic extracellular solution, which caused the VRAC channels to open. The VRAC currents gradually increased in response to the hypotonic environment until they reached a plateau, which we referred to as *I*_0_ (pA). (Figure 2A–C). VRAC currents exhibited a classic inactivation feature under high positive potentials in the full voltage recording mode (Figure 1E). Furthermore, VRAC currents showed typical outward rectification features under ramp protocol from −100 mV to +100 mV (Figure 2A–C). To investigate the effect of syringin on VRAC currents, HEK293 cells were perfused with hypotonic extracellular solution containing different concentrations of syringin (6.25, 25, and 100 μM). The VRAC currents rapidly decreased and reached a plateau in a dose‐dependent manner upon the perfusion of syringin‐containing hypotonic solution, and these currents were referred to as *I* (pA). (Figure 2A–C). Full voltage test showed that 100 μM syringin inhibited VRAC currents under every full potential (from −100 to +100 mV) (Figure 2E). The mean ratio of *I*/*I*_0_ for 6.25, 25, and 100 μM syringin on VRAC currents was 0.95 ± 0.01 (*n* = 4), 0.79 ± 0.02 (*n* = 5), and 0.61 ± 0.02 (*n* = 5), respectively. (Figure 2F). We also tested the effect of the selective VRAC channel inhibitor, DCPIB (20 μM), on the hypotonic solution‐induced currents, which almost completely inhibited the VRAC currents. (Figure 2D,F). Taken together, these results suggested that syringin suppressed VRAC channels in a dose‐dependent relationship. Our study first identified syringin as a VRAC channels blocker.

### 3.2 The predicted interaction between syringin and the VRAC protein

DCPIB is a specific and potent inhibitor of VRAC channels. Kern et al. presented single‐particle cryo‐electron microscopy structures of LRRC8A in complex with the inhibitor DCPIB reconstituted in lipid nanodiscs.^30^ They described that the DCPIB plugged the channel like a cork in a bottle‐binding in the extracellular selectivity filter and sterically occluding ion conduction. To investigate the potential interaction mode of syringin and VRAC channels, we set the grid box of VRAC channel based on the location of DCPIB binding on VRAC protein described by Kern et al. Syringin docked with VRAC protein in the region of the grid box. The docking work of syringin and VRAC channel was produced by AutoDock Vina 1.5.6 software. The calculation results indicated that syringin has an affinity for VRAC protein. In total, nine combinations of syringin and VRAC were calculated by the software. Among them, the best conformation was predicted to have a binding affinity of −6.8 kcal/mol (Table 1).

**TABLE 1:** Docking parameters of syringin and volume regulated anion channel protein.

Mode	Affinity (kcal/mol)	Dist from the best mode	
		rmsd l.b.	rmsd u.b.
1	−6.8	0.000	0.000
2	−6.7	2.660	5.483
3	−6.7	2.275	4.841
4	−6.7	2.212	5.915
5	−6.7	3.361	6.498
6	−6.7	2.246	5.970
7	−6.6	2.359	6.056
8	−6.5	3.389	5.887
9	−6.3	2.542	6.775

**FIGURE 3:**
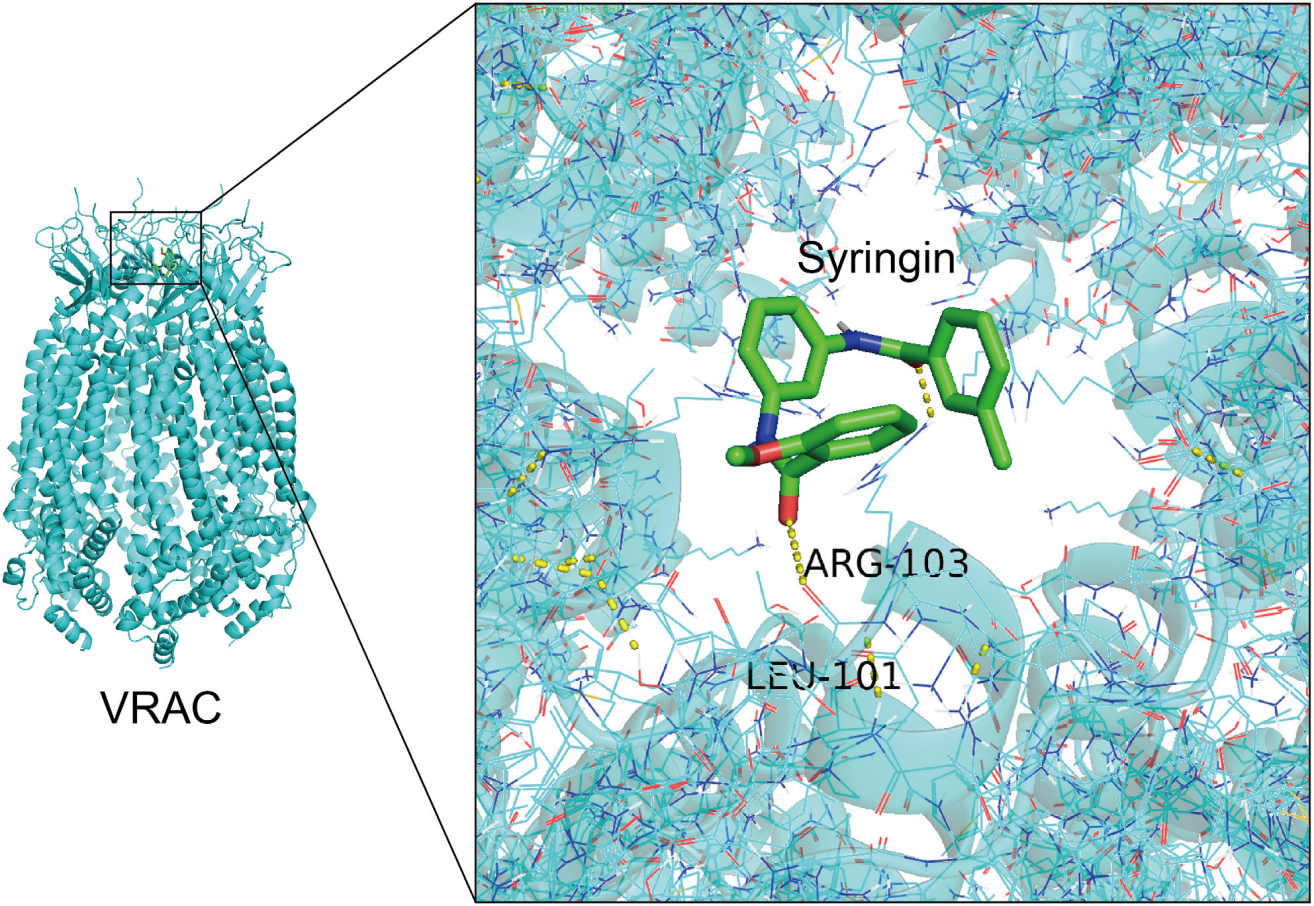
Binding mode (mode 1) of syringin and volume regulated anion channel (VRAC) channel.

We used PyMOL software to predict the interacting amino acid residues of VRAC protein. The computational prediction indicated that syringin may interact with the VRAC protein in the extracellular domain and share the same binding site as DCPIB, arginine 103, and have another binding site at leucine 101. (Figure 3).

## 4 DISCUSSION

In conclusion, our study investigated the effects of syringin on endogenous VRAC currents induced by hypotonic extracellular solution in HEK293 cells. Syringin showed a dose‐dependent inhibition of VRAC current. Additionally, in silico calculations indicated that syringin may have a high affinity for LRRC8/VRAC protein, suggesting that syringin may have a potential direct binding effect on VRAC channels. Given that VRAC channels mediate the osmotic excitotoxic substances efflux from astrocytes in ischemic injury, we propose that syringin may have the potential to rescue swollen astrocytes in ischemic injury by inhibiting VRAC channels, which could reduce the release of glutamate and potentially decrease neurotoxic damage to neurons. Syringin has been shown to possess anti‐oxidant properties, as evidenced by its ability to inhibit reactive oxygen species (ROS) levels in the brains of APP/PS1 transgenic mice,^31^ reduce mtROS induced by Aβ_(25–35),_ ^32^ decrease ROS in diabetic rats, and inhibit ROS induced by H_2_O_2_ in H9c2 cells.^33^ Interestingly, stimulation of mitochondrial‐mediated apoptosis by staurosporine rapidly activates VRAC and generates ROS within a similar time frame.^34^ Given these findings, we are interested in whether the anti‐ROS effects of syringin are related to its modulation of VRAC channel activity. Although the results have shown the calculative potential binding sites of syringin and LRRC8A proteins, this is still a putative model. In future studies, we plan to explore the effect of syringin on LRRC8A‐R103A mutant currents to determine whether syringin has no or less inhibition on mutant LRRC8A currents. Further studies are needed to refine our results, such as constructing an IC_50_ curve for syringin and testing its specificity for VRAC channels.

Due to the critical role of VRAC channels in cell apoptosis, insulin secretion, anti‐viral immunity, and particularly in cerebral ischemia, there is a pressing need to develop regulators of VRAC channels. To date, DCPIB is widely recognized as a specific and potent inhibitor of VRAC channels. However, accumulating evidence demonstrates that DCPIB not only inhibits the VRAC channels but also suppresses the other proton pumps or ion channels such as H+/K+ ATPase, connexin hemichannels, K2P channels, including TRESK, TASK1, TASK3. DCPIB even displays the agonist function toward TREK1, TREK2, and TRAAK channels (all belonging to K2P channels).^35^, ^36^, ^37^, ^38^ Besides that, 6u is a newly synthesized VRAC channel inhibitor derived from the structure of DCPIB, which shows strong inhibition to VRAC channels (IC_50_: 7.11 ± 0.94 μM). Interestingly, 6u also acts as a dual inhibitor, as it is able to inhibit TREK1 channels.^21^ In summary, VRAC channels lack selective regulators, which limits further research on the VRAC channels. For this reason, we need to discover regulators with another whole new structure.

In the current study, we first characterize syringin as an inhibitor for the VRAC channels. Syringin is the first VRAC regulator with a monosaccharide derivative structure, which provides a perspective for future VRAC channel regulator development. We hope that the inhibitory effect of syringin on VRAC channels will provide a theoretical basis for the inhibitory mechanism of syringin on brain edema.

## AUTHOR CONTRIBUTIONS

Participated in research design and performance: Ziwei Xu, Wei Yao, Minyan Liang, Song Wang, Longhui Lu, Jingjing Wang, and Na Zhu. Wrote or contributed to the writing of the manuscript: Ziwei Xu and Liping Huang.

## FUNDING INFORMATION

We gratefully acknowledge the financial support for this study provided in part by the National Natural Science Foundation of China (82060759), the Jiangxi University of Chinese Medicine Graduate Student Innovation Special Fund (at the University Level) (JZYC22B03), and the Jiangxi University of Chinese Medicine Science and Technology Innovation Team Development Program (CXTD22007).

## DISCLOSURE

All authors declare that they have no conflict of interest.

## ETHICS APPROVAL STATEMENT

None.

## Data Availability

Raw data can be made available upon request from the authors.
